# Liraglutide use in congenital generalized lipodystrophy: glycemic and metabolic outcomes

**DOI:** 10.3389/fendo.2026.1912138

**Published:** 2026-09-22

**Authors:** Jéssica S. Araújo, Natália P. Boris, Fábia Karine de M. Lopes, Amanda C. Flor, Liz Marjorie B.F. Leite, Lana Lívia P. Linard, Isabella C. Bezerra, Janio Emanuel A. Cavalcante, Edson Tanaka, Fabiano Sandrini, Virgínia O. Fernandes, Renan M. Montenegro Junior

**Affiliations:** 1 Brazilian Group for the Study of Inherited and Acquired Lipodystrophies (BRAZLIPO), Fortaleza, CE, Brazil; 2 Clinical Research Unit, Walter Cantídio University Hospital, Federal University of Ceará/Empresa Brasileira de Serviços Hospitalares - Brazilian Hospital Services Company (EBSERH), Fortaleza, CE, Brazil; 3 Clinical and Toxicological Analysis Laboratory – Prof. PhD. Eurico Litton Pinheiro de Freitas, Federal University of Ceará, Fortaleza, CE, Brazil; 4 Department of Endocrinology, University Hospital of Western Paraná, Cascavel, PR, Brazil; 5 Department of Community Health, Federal University of Ceará, Fortaleza, CE, Brazil; 6 Department of Clinical Medicine, Federal University of Ceará, Fortaleza, CE, Brazil

**Keywords:** congenital generalized lipodystrophy, diabetes mellitus, glucagon-like peptide-1 receptor agonists, lipodystrophy, liraglutide

## Abstract

**Objective:**

Evidence on glucagon-like peptide 1 receptor agonists (GLP-1RAs) in lipodystrophies is limited. We evaluated the effects of liraglutide on metabolic control and satiety in patients with congenital generalized lipodystrophy (CGL).

**Methods:**

In this case series, patients with CGL received liraglutide 1.8 mg/day for 12 weeks. Metabolic parameters, satiety, and body composition were assessed at baseline, week 12, and after a 12-week post-treatment follow-up (week 24). Liver imaging was performed at baseline and week 12.

**Results:**

Eight patients were enrolled (aged 24–45 years; 75% female), and three discontinued treatment due to gastrointestinal adverse events during the dose-escalation phase. Among the five participants who completed the 12-week intervention, liraglutide significantly reduced HbA1c (median change, −2.5%; p=0.012) and total daily insulin dose (−20%; p=0.042), whereas the reduction in triglyceride levels was not statistically significant. Twelve weeks after liraglutide discontinuation, median HbA1c and body weight increased by 2.2% (p=0.026) and 3.7% (p=0.001), respectively. Triglyceride levels increased in 80% of participants, with a median increase of 421.5 mg/dL (p=0.334). Insulin requirements also increased overall: one participant resumed insulin therapy, while the remaining participants increased their insulin dose by a median of 22% (p=0.024).

**Conclusions:**

This first study of liraglutide in CGL provides an exploratory signal of improved glycemic control and reduced insulin requirements, but treatment tolerability remains a limitation. Larger, controlled, and longer-term studies are needed to confirm these effects and assess potential benefits on satiety, body composition, and liver disease.

## Graphical Abstract

**Figure:**
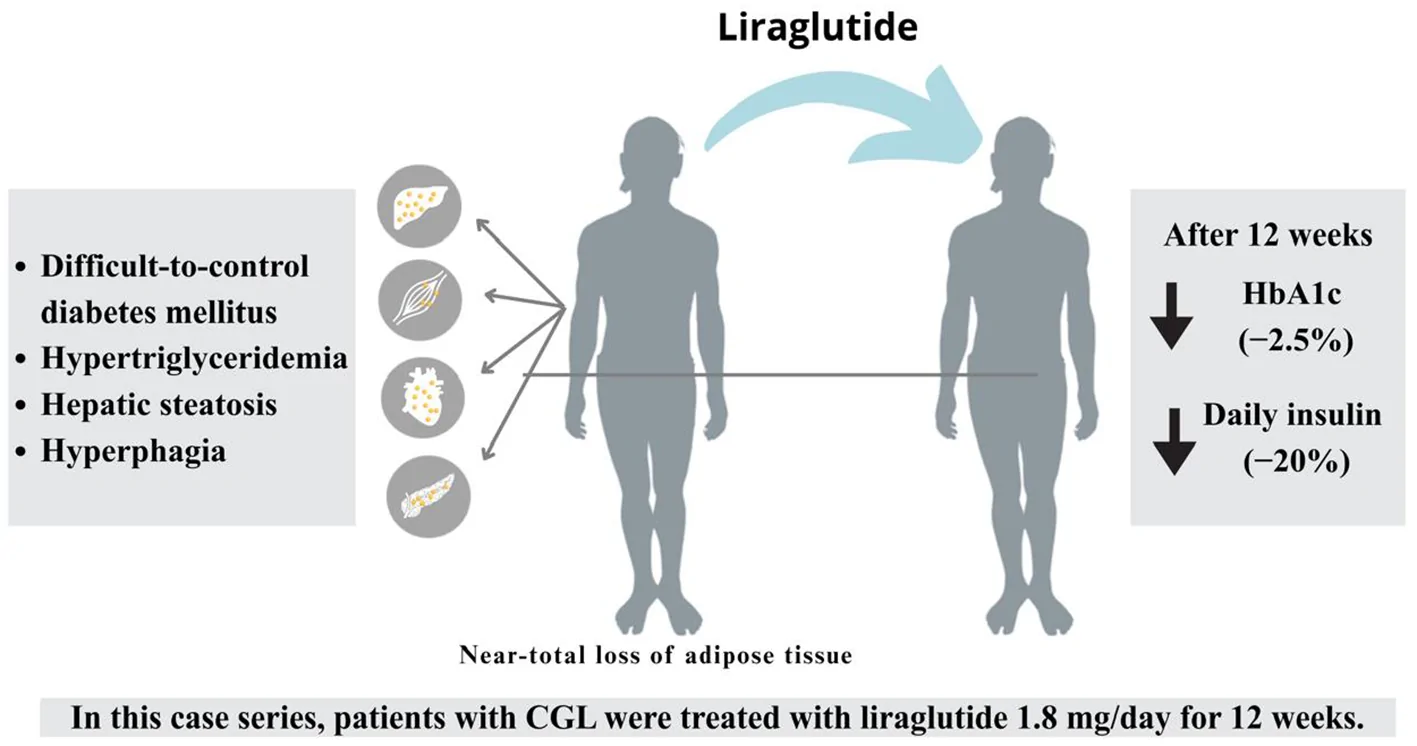

## Introduction

Lipodystrophies comprise a heterogeneous and rare group of disorders characterized by complete or partial loss of subcutaneous adipose tissue, resulting in ectopic fat accumulation in organs such as the pancreas, muscle, and liver, which may lead to severe metabolic complications (1).

Congenital generalized lipodystrophy (CGL) presents with near-total loss of adipose tissue, hyperphagia secondary to extremely low leptin levels (2), difficult-to-control diabetes mellitus, hypertriglyceridemia, and hepatic steatosis, all of which significantly impact survival (3). There are four recognized clinical–molecular subtypes of CGL (types 1–4), caused by mutations in *AGPAT2, BSCL2, CAV1, and PTRF*, respectively, with approximately 95% of cases attributable to mutations in *AGPAT2* and *BSCL2* (4).

Metreleptin is currently the only specific therapy available for this condition; however, its clinical use is limited by restricted availability in many countries, including Brazil (5, 6). Additional therapeutic strategies are therefore needed to address the severe metabolic complications associated with the disease. In this context, glucagon-like peptide-1 receptor agonists (GLP-1RAs), particularly liraglutide, have emerged as potential adjunctive metabolic therapies rather than substitutes for leptin replacement and may warrant investigation as therapeutic options in settings where metreleptin is unavailable or access is limited, owing to their well-established effects in obesity and type 2 diabetes mellitus, including improved glycemic control and insulin sensitivity, favorable effects on lipid metabolism, delayed gastric emptying, reduced appetite, and decreased adiposity (7).

Diabetes mellitus is a common manifestation of lipodystrophy, with an estimated prevalence of approximately 58.3% (8), and contributes substantially to its associated complications. In patients at high cardiovascular risk, GLP-1RAs are recommended as first-line therapy due to their proven cardiovascular benefits (9). Overall, individuals with lipodystrophies appear to be at particularly high risk for atherosclerotic cardiovascular disease (ASCVD), largely driven by the high prevalence of dyslipidemia and diabetes. Therefore, aggressive management of these metabolic complications is warranted to mitigate ASCVD risk in this population (10).

Evidence regarding the use of GLP-1RAs in patients with lipodystrophies remains scarce, being limited to case reports, a retrospective cohort involving 14 individuals with familial partial lipodystrophy (FPLD) (11–14), and an experimental study in *Bscl2*-null (seipin knockout) mice demonstrating improvements in appetite control, glycemic homeostasis, and hepatic fibrosis markers (15). A more recent observational cohort study has also evaluated the use of tirzepatide, a dual GLP-1 and glucose-dependent insulinotropic polypeptide (GIP) receptor agonist (GIP/GLP-1RA), in patients with lipodystrophy (16). To our knowledge, this is the first study evaluating the effects of liraglutide on metabolic parameters and satiety in patients with congenital generalized lipodystrophy.

## Materials and methods

A case series of liraglutide treatment was conducted from January 2024 to April 2025 at the coordinating center of the Brazilian Study Group on Hereditary and Acquired Lipodystrophy (BRAZLIPO). A total of 31 patients with CGL were registered at the site, including 11 adults under regular follow-up, 3 adults living outside the region, 11 children/adolescents, and 6 patients who had died. This study protocol was approved by the Ethics Committee of the Walter Cantídio University Hospital of the Federal University of Ceará (Ethics approval ID: CAAE: 76555123.5.0000.5045) and was performed in accordance with the principles of the Declaration of Helsinki. Written informed consent was obtained from the patients.

Eligible participants were adults aged 18–50 years with a genetically confirmed diagnosis of CGL. Exclusion criteria included pregnancy or lactation, renal dysfunction with estimated glomerular filtration rate (eGFR, CKD-EPI) <15 mL/min/1.73 m², body mass index (BMI) <18.5 kg/m², prior history of pancreatitis, severe gastroesophageal reflux disease and refusal to participate.

Participants received liraglutide (6 mg/mL) for 12 weeks. Liraglutide was initiated at 0.6 mg/day and titrated weekly to 1.2 mg/day and subsequently to 1.8 mg/day, provided it was well tolerated, according to the approved prescribing information. Given inadequate glycemic control despite ongoing basal therapy, the 1.8 mg/day dose was set as the target to optimize glycemic control. No additional changes were made to concomitant therapy, except for insulin dose adjustments or discontinuation according to self-monitored capillary blood glucose targets of 80–130 mg/dL in the fasting state and <180 mg/dL 2 hours postprandially. Weekly online visits were conducted to ensure medication adherence. Monthly visits assessed treatment response, medication adjustments, adverse effects, and injection technique. No new dietary recommendations were provided beyond those already in use.

Baseline data included demographic characteristics (age, biological sex), CGL subtype, age at diagnosis, diabetes duration, presence of microvascular or macrovascular complications, and metabolic dysfunction–associated steatotic liver disease (MASLD), defined as hepatic steatosis on ultrasound. Changes in body weight, glycated hemoglobin (HbA1c), triglyceride levels, FIB-4 liver fibrosis score, and total daily insulin requirement were assessed at baseline, after liraglutide treatment, and at 12 week follow up after treatment discontinuation (24 weeks). Hepatic steatosis and fibrosis were evaluated using abdominal ultrasound and transient elastography (FibroScan) at baseline and at the end of treatment when available.

Additionally, satiety was assessed at baseline and after 12 and 24 weeks using a visual analog scale (VAS) (17), administered following a 12-hour overnight fast. The instrument comprised eight items evaluating hunger, fullness, satiety, and prospective food consumption. Items assessing hunger and prospective food consumption, for which higher scores indicate greater appetite and lower satiety, were reverse-scored before calculating the total score. Each item was scored from 0 to 10 (total score range, 0–80), with higher total scores indicating greater satiety. Whole-body dual-energy X-ray absorptiometry (DEXA) was performed at baseline and at 12 and 24 weeks, when available, to assess body composition. Analyses were performed using all available data for each outcome; therefore, the number of participants included varied according to data availability. Continuous variables were expressed as median (minimum–maximum). Changes in variables over time were analyzed using the Friedman test among the five participants who completed the 12-week intervention, adopting a significance level of 5%.

## Results

Eight patients were enrolled in the study. Baseline characteristics are summarized in **Table 1**. The participants exhibited a high cardiometabolic risk profile, characterized by long-standing diabetes requiring insulin therapy, severe insulin resistance, dyslipidemia, and a high burden of chronic diabetes-related complications. None of the participants had prior or current exposure to metreleptin. Gastrointestinal adverse effects, including nausea, vomiting, postprandial fullness, and diarrhea, were reported, leading to treatment discontinuation in three patients during the dose-escalation phase (cases 1-3). Discontinuation occurred in case 1 during the second week of treatment at a dose of 1.2 mg/day, and in cases 2 and 3 during the first week at a dose of 0.6 mg/day. The remaining patients (cases 4–8) reached the target liraglutide dose of 1.8 mg once daily, with overall good treatment tolerability.

**Table 1:** Baseline characteristics of CGL patients.

Patient n°	1	2	3	4	5	6	7	8
Age (years)	28	35	45	44	42	40	35	24
Sex	Female	Female	Female	Male	Female	Female	Female	Male
Subtype	CGL1	CGL1	CGL1	CGL1	CGL1	CGL1	CGL1	CGL2
Pathogenic variants	*AGPAT2* c.589-2A>G (p.Gln196fs*228); c.646A>T (p.Lys216*), both heterozygous	*AGPAT2* c.369_372delGCTC (p.Leu124Serfs*26); c.589-2A>G (p.Gln196fs*228), both heterozygous	*AGPAT2* c.299G>A (p.Ser100Asn); c.493-1G>C (p.Leu165_Gln196del), both homozygous	*AGPAT2* c.366-588 + 534del (p.Gly106fs*188), homozygous	*AGPAT2* c.589-2A>G, homozygous	*AGPAT2* c.646A>T (p.Lys216*), homozygous	*AGPAT2* c.366-588 + 534del (p.Gly106fs*188), homozygous	*BSCL2* c.301_302insAA (p.Met101Lysfs11); c.325dupA (p.Thr109Asnfs5), both heterozygous
BMI (kg/m²)	18.7	24	24.3	24	20.8	22.6	23.3	24.5
Age at diagnosis of CGL (years)	28	0.25	38	41	29	28	3	1.75
Diabetes duration (years)	27	22	17	27	12	24	24	11
Comorbidities	Diabetes with retinopathy, neuropathy and DKD, MASLD	Diabetes with retinopathy, neuropathy and DKD, CAD, PAOD	Diabetes with retinopathy, MASLD	Diabetes with retinopathy, CAD, MASLD	Diabetes, MASLD	Diabetes with retinopathy, neuropathy and DKD, MASLD	Diabetes with retinopathy and neuropathy, MASLD	Diabetes with DKD, PAOD, MASLD
Antidiabetic drugs in use	Insulin 2, 6 IU/kg/day	Insulin 4, 8IU/kg/day	Metformin 2 gram/day + insulin 1, 1 IU/kg/day	Metformin 1 gram/day + insulin 0.5 IU/kg/day	Metformin 1 gram/day + insulin 1, 8 IU/kg/day	Metformin 2 gram/day + insulin 2, 7 IU/kg/day	Metformin 2 gram/day + insulin 2, 1 IU/kg/day	Metformin 2 gram/day + insulin 1, 4 IU/kg/day

CGL, congenital generalized lipodystrophy; *AGPAT2*, 1-acylglycerol-3-phosphate O-acyltransferase 2; *BSCL2*, Berardinelli-Seip congenital lipodystrophy type 2 (seipin); BMI, body mass index; DKD, diabetic kidney disease; MASLD, metabolic dysfunction-associated steatotic liver disease; CAD, coronary artery disease; PAOD, peripheral arterial occlusive disease.

Among the five participants who completed the 12-week intervention, liraglutide was associated with improvements in glycemic control and insulin requirements. After 12 weeks of therapy, the median HbA1c decreased by 2.5% (27 mmol/mol; range, 1.1–3.1% [12–34 mmol/mol]; p = 0.012), total daily insulin dose decreased by 20% (range, 0–100%; p = 0.042), and triglyceride levels decreased by a median of 127 mg/dL (range, 64–1171 mg/dL), although this change was not statistically significant (p = 0.350) (**Table 2**). One participant (Case 4) experienced hypoglycemia, leading to discontinuation of insulin therapy.

**Table 2:** Metabolic parameters at baseline (0) and after 12 weeks of liraglutide use (12w), and percentage of changes from baseline observed.

Patient n°	4	5	6	7	8	Median (min.-max.)	*p*
HbA1c (0) (%) (n=5)	8.6	10	8.8	8.9	10.6	8.9 (8.6-10.6)	
HbA1c (12w) (%) (n=5)	7.3	6.9	6.3	6.4	9.5	6.9 (6.3-9.5)	
Change in HbA1C (%) (n=5)	-1.3	-3.1	-2.5	-2.5	-1.1	-2.5 (1.1-3.1)	**0.012**
TG (0) (mg/dL) (n=5)	435	1267	182	382	242	382 (182-1267)	
TG (12w) (mg/dL) (n=5)	210	96	118	255	157	157 (96-255)	
Change in TG (mg/dL) (n=5)	-225	-1171	-64	-127	-85	-127 (64-1171)	0.350
Change in total daily insulin dose (%) (n=5)	-100	-30	-20	-15	0	-20 (0-100)	**0.042**

TG, Triglycerides.

Values ​​in bold indicate a statistically significant difference (p<0.05).

Regarding body weight, two patients (cases 6 and 7) presented weight loss and three patients (case 4, 5 and 8) weight gain (p=0.990). Body composition assessed by DEXA showed heterogeneous individual responses. No consistent changes were observed in fat mass index (FMI) and relative skeletal muscle index (RSMI) after 12 weeks of treatment (**Table 3**). Satiety, assessed using a visual analog scale (VAS), increased in two patients (from 35 to 40 and from 30 to 52 in cases 5 and 6, respectively), decreased in two patients (cases 4 and 8), and remained unchanged in one patient (case 7) (**Table 3**).

**Table 3:** Body composition, satiety, and liver-related parameters assessed at baseline (0), after 12 weeks of liraglutide treatment (12w), and at 12-week follow-up after treatment discontinuation (24w) in patients with CGL.

Patient n°	4	5	6	7	8	Median (min.-max.)	*p*
Body composition							
Body weight (0) (kg) (n=5)	64.8	54.5	61.7	65	76.1	64.8 (54.5-76.1)	
Body weight (12w) (kg) (n=5)	65.3 (+0.8%^†^)	57.2 (+5%^†^)	59.6 (-3.4%^†^)	63.2 (-2.7%^†^)	76.2 (+0.2%^†^)	63.2 (57.2-76.2^†^)	0.990^†^
Body weight (24w) (kg) (n=5)	68.4 (+4.8% ^§^)	59 (+3.2% ^§^)	61.7 (+3.6% ^§^)	65.5 (+3.7% ^§^)	79 (+3.7% ^§^)	65.5 (59-79) +3.7% (3.2- 4.8%^§^)	**0.001** ^§^
% Total fat (0) (n=4)	10.5	-	9.1	11.8	9.8	10.1 (9.1-11.8)	
% Total fat (12w) (n=4)	10.6	-	8.5	12.2	8.9	9.7(8.5-12.2)	0.50^†^
%Total fat (24w) (n=4)	11.1	-	8.9	11.9	9.1	10.1(8.9-11.9)	0.37^§^
FMI (0) (n=4)	2.43	-	2.04	2.75	2.40	2.41(2, 04-2, 75)	
FMI (12w) (n=4)	2.55	-	1.90	2.75	2.20	2.37 (1.90-2.75)	0.50^†^
FMI (24w) (n=4)	2.87	-	1.99	2.75	2.27	2.51 (1.99-2.87)	0.26^§^
RSMI (0) (n=4)	8.12	-	8.31	8.62	7.98	8.21(7.98-8.62)	
RSMI (12w) (kg/m²) (n=4)	8.38	-	8.37	8.36	8.25	8.36(8.25-8.38)	0.125^†^
RSMI (24w) (kg/m²) (n=4)	8.89	-	8.17	8.40	8.24	8.32(8.17-8.89)	0.875^§^
Satiety							
VAS score (0) (n=5)	80	35	30	39	50	39 (30-80)	
VAS score (12w) (n=5)	30	40	52	39	40	40 (30-52)	0.76^†^
VAS score (24w) (n=4)	25	-*	38	37	45	37.5 (25-45)	0.25^§^
Liver-related parameters							
FIB-4 (0) (n=5)	0.63	1.45	0.54	0.58	1.05	0.63 (0.54-1.45)	
FIB-4 (12w) (n=5)	0.69	1.10	0.71	0.67	0.82	0.71 (0.67-1.10)	0.868^†^
FIB-4 (24w) (n=5)	1.65	1.77	0.67	1.11	0.96	1.11(0.67-1.77)	0.148^§^
Fatty liver disease on AU (0) (n=5)	Mild	Mild	Moderate	Moderate	Moderate		
Fatty liver disease on AU (12w) (n=5)	Mild	Mild	Mild	Mild	Moderate		
CAP to Fibroscan (0) (dB/m) (n=2)	-	-	-	304 (S3)	250 (S1)		
CAP to Fibroscan (12w) (dB/m) (n=2)	-	-	200 (S0)	-	289 (S3)		
LSM to Fibroscan (0) (kPa) (n=2)	-	-	-	5.6 (F0)	23 (F4)		
LSM to Fibroscan (12w) (kPa) (n=2)	-	-	3.9 (F0)	-	25.4 (F4)		

Body weight, body composition (DEXA), satiety (VAS) and FIB-4 were assessed at baseline (0), week 12 (12w), and week 24 (24w). Abdominal ultrasound and FibroScan were performed at baseline and week 12. DEXA measurements were available for four participants at all study visits. FibroScan measurements were available for two participants at baseline and week 12.

FMI, fat mass index; RSMI, relative skeletal muscle index; VAS, visual analog scale of satiety; FIB-4, fibrosis-4 index; AU, abdominal ultrasound**;** CAP, controlled attenuation parameter; LSM, liver stiffness measurement; S0, steatosis grade 0; S1, steatosis grade 1; S3, steatosis grade 3; F0, fibrosis grade 0; F4: fibrosis grade 4.

†From baseline (0) to week 12 (12w).

^§^ From week 12 (12w) to week 24 (24w).

*Data not available.

Values ​​in bold indicate a statistically significant difference (p<0.05).

The FIB-4, abdominal ultrasound, and FibroScan data are presented in **Table 3**. In cases 5 and 8, FIB-4 scores decreased from 1.45 to 1.1 and from 1.05 to 0.82, respectively (p=0.868), attributable to increased platelet counts (from 151, 000 to 241, 000 in case 5 and from 73, 000 to 111, 000 in case 8), consistent with the reduction in spleen size observed on abdominal ultrasound (from 14.6 to 12.8 cm in case 5 and from 15.4 to 13.8 cm in case 8). However, case 8 had established cirrhosis and portal hypertension at baseline, representing an important source of clinical heterogeneity and a potential confounding factor in the interpretation of hepatic outcomes. In this case, hepatic steatosis progressed from grade 1 to grade 3, as indicated by an increase in controlled attenuation parameter (CAP) from 250 to 289 dB/m on FibroScan, while the fibrosis stage remained stable at F4. Regarding the quantification of hepatic fat by ultrasound, three patients (cases 4, 5, and 8) remained stable, whereas two patients (cases 6 and 7) showed a reduction in the degree of steatosis from moderate to mild.

After 12 weeks of liraglutide discontinuation, all patients experienced increases in HbA1c and body weight, with median changes of 2.2% (24 mmol/mol; range 0.8–3.5% [9–38 mmol/mol]; p=0.026) and 3.7% (range 3.2–4.8%; p=0.001), respectively. Triglyceride levels increased in 80% of patients, with a median change of 421.5 mg/dL (range 76–1649 mg/dL; p=0.334). Regarding insulin therapy, patient 4 resumed insulin at 0.4 U/kg/day and the remaining patients experienced a median dose increase of 22% (range 12–50%; p=0.024). In cases 4, 5, and 8, platelet counts decreased, associated with elevations in FIB-4 scores from 0.69 to 1.65, 1.10 to 1.77 and 0.82 to 0.96, respectively. In patient 7, an increase in FIB-4 from 0.67 to 1.11 was observed, accompanied by an increase in transaminases. In patient 6, there was a decrease in FIB-4 from 0.71 to 0.67 (p=0.148). **Figure 1** shows changes in metabolic parameters, total daily insulin dose, and body weight at baseline, after 12 weeks of liraglutide use and after 12 weeks of drug discontinuation.

**Figure 1:**
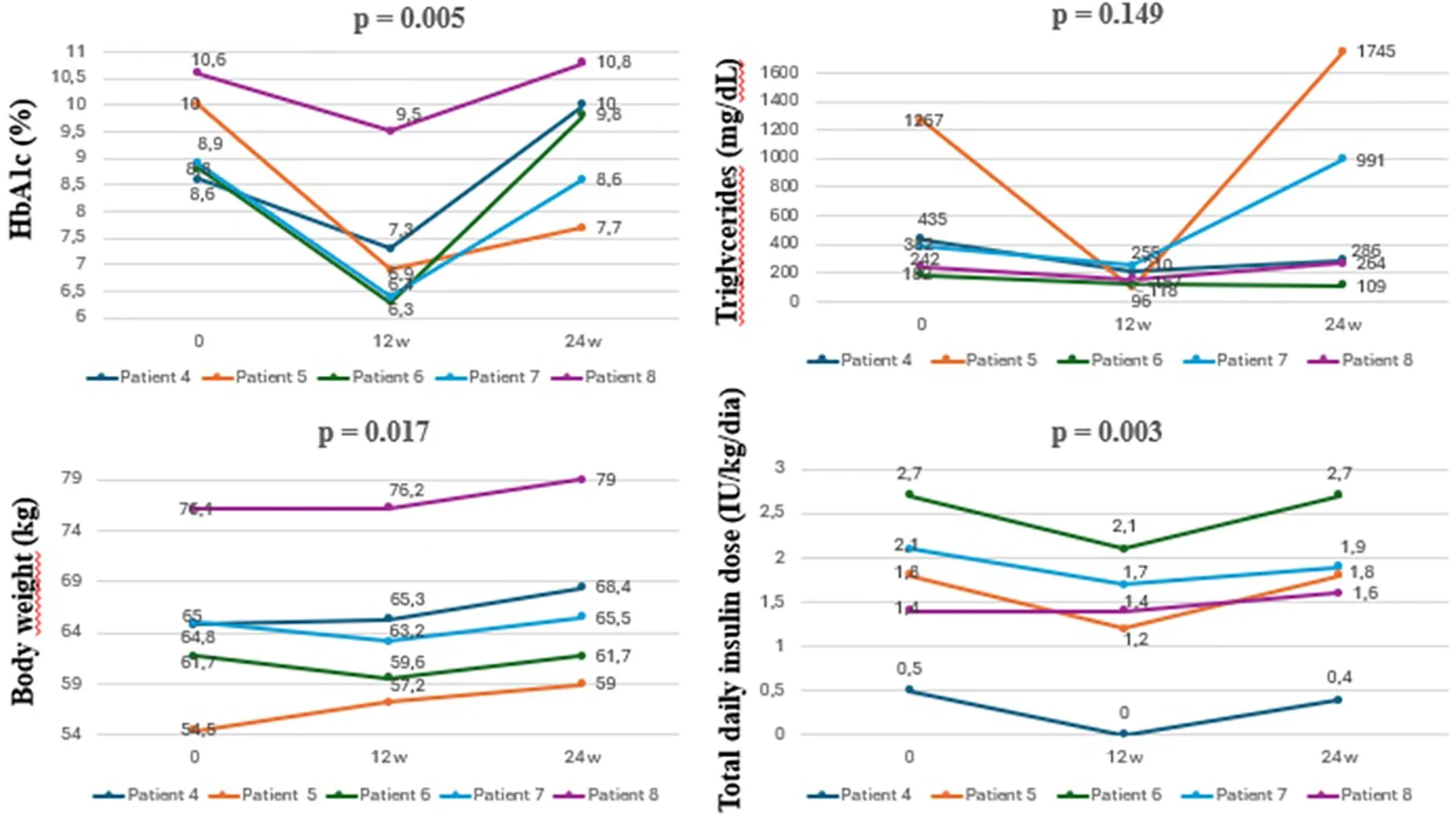
Changes in metabolic parameters, total daily insulin dose, and body weight at baseline (0), after 12 weeks of liraglutide treatment (12w), and at 12-week follow-up after treatment discontinuation (24w) in patients with CGL.

## Discussion

This study evaluated the effects of the GLP-1RA liraglutide on metabolic parameters, body composition, satiety, and adverse event profile in eight patients with CGL. To our knowledge, this represents the largest reported experience with GLP-1RA in CGL to date.

The cardiovascular benefits of GLP-1RAs, characterized by reductions of major adverse cardiovascular events (MACE), cardiovascular mortality, and all-cause mortality in patients with type 2 diabetes, have been consistently demonstrated in large randomized clinical trials and meta-analyses (18, 19). These findings support current guideline recommendations for the use of GLP-1RA as first-line therapy in patients at high and very high cardiovascular risk (9). Although specific guideline recommendations are currently lacking for patients with CGL, the high cardiometabolic burden associated with this condition provides a strong rationale for investigating GLP-1RAs in this population.

Furthermore, GLP-1RAs enhances pharmacological management of diabetes and obesity through both central and peripheral mechanisms. The reduction in caloric intake via satiety induction may mimic one of the benefits of leptin replacement in patients with lipodystrophy (20–22). It was recently demonstrated that the central anorexigenic effect of leptin is mediated by neurons co-expressing GLP-1 and the leptin receptor in the dorsomedial hypothalamus of rodents and primates (23, 24). In these studies, combined administration of leptin and GLP-1 receptor agonists resulted in more pronounced reductions in food intake, greater weight loss, and preservation of lean mass.

In clinical practice, metreleptin in combination with dietary intervention remains the first-line therapy for patients with CGL (25). This recommendation is supported by a study that included 66 patients with generalized lipodystrophy (GL) treated for 12 months, demonstrating significant reductions in triglyceride levels (from approximately 1, 329 mg/dL to 531 mg/dL; p = 0.001) and HbA1c (from 8.6% [70 mmol/mol] to 6.4% [46 mmol/mol]; p < 0.001) (5). Nevertheless, evidence regarding the combined use of GLP-1RAs and leptin replacement therapy in patients with CGL is currently lacking.

The metabolic efficacy of GLP-1RAs in FPLD has been described in case reports and a retrospective cohort of 14 patients, showing weight reduction of 4.0%, BMI decrease of 4.1%, triglyceride reduction of 16.7%, and HbA1c decrease of 0.5% (5 mmol/mol) (10–12). A previous report described improved glycemic control with an oral GLP-1RA in a patient with GL and persistent hyperglycemia despite metreleptin therapy and high-dose insulin, allowing insulin discontinuation (14). Ten et al. (26) reported two patients with CGL subtype 1 (CGL1) treated with weekly tirzepatide 15 mg who achieved glycemic control without additional insulin therapy within 1–3 weeks. More recently, an observational study in a heterogeneous cohort of 17 patients with lipodystrophy (14 with FPLD, 1 with atypical FPLD, and 2 with acquired generalized lipodystrophy) demonstrated a favorable tolerability profile (14). Among FPLD patients, absolute HbA1c decreased by 1.1% (12 mmol/mol; p < 0.001), the daily insulin dose decreased by 101 IU (p = 0.003), BMI decreased by 1.9 kg/m² (p = 0.008), and triglycerides were decreased by a median of 35% (p = 0.003). Our findings on reductions in HbA1c, triglycerides, and total daily insulin are consistent with these prior reports.

An illustrative finding was observed in Case 4, in whom recurrent hypoglycemia during liraglutide treatment led to complete discontinuation of insulin therapy. At 12-week follow-up after treatment discontinuation, insulin was reintroduced at a dose of 0.4 U/kg/day because glycemic control deteriorated after liraglutide withdrawal. Although this observation is limited to a single patient, it highlights both the marked insulin-sparing effect of liraglutide and the apparent loss of this metabolic benefit after treatment discontinuation, even in a patient who had achieved insulin independence.

As previously reported in patients with diabetes treated with GLP-1RAs or dual GIP/GLP-1RA (27–34), an increase in body weight following treatment discontinuation was observed in the present study. However, because no significant overall weight loss occurred during liraglutide treatment, this finding should not be interpreted as weight regain or a rebound effect. The mechanisms underlying the observed increase in body weight after treatment discontinuation remain uncertain and may involve physiological responses to treatment withdrawal, although this cannot be established from our data.

Importantly, satiety assessed by a visual analog scale, body composition measured by DEXA, and liver involvement assessed by FIB-4, abdominal ultrasound, and FibroScan have not previously been evaluated in patients with CGL. In individuals with diabetes, GLP-1RAs have been associated with attenuation of liver fibrosis progression over follow-up periods of approximately 24–72 weeks (35–38), as well as improvements in body composition after around 48 weeks (39). A noteworthy finding was the progression of hepatic steatosis in Case 8, with controlled attenuation parameter (CAP) values increasing from S1 (250 dB/m) at baseline to S3 (289 dB/m) after treatment, while liver stiffness remained stable at F4. Furthermore, this participant had advanced liver disease with established cirrhosis and portal hypertension, conditions that may influence both the natural history of MASLD and the response to pharmacological interventions. Because MASLD in CGL is driven by profound adipose tissue deficiency and leptin deficiency rather than obesity, the hepatic effects of GLP-1RAs may differ from those observed in more common metabolic disorders. Although this isolated finding does not establish a causal relationship between liraglutide and worsening hepatic steatosis, it underscores the heterogeneity of treatment responses and highlights the need for longer prospective studies incorporating serial liver imaging to better characterize the hepatic effects of GLP-1RAs in patients with CGL. In addition, the interpretation of non-invasive liver fibrosis markers in CGL warrants careful consideration.

Although no statistically significant change in FIB-4 was observed during liraglutide treatment, this index warrants cautious interpretation in patients with CGL. Although FIB-4 is a widely used non-invasive marker of liver fibrosis, it has not been specifically validated in this population, particularly in the presence of portal hypertension and splenomegaly. In our study, the apparent reduction in FIB-4 observed in Cases 5 and 8 was primarily driven by increased platelet counts, likely reflecting improvement in hypersplenism secondary to reduced splenomegaly rather than regression of hepatic fibrosis. Because platelet count is incorporated into the FIB-4 equation and may be influenced by extrahepatic factors in advanced CGL, changes in this index should be interpreted cautiously. In this setting, liver stiffness measurement by transient elastography may provide a more direct assessment of hepatic fibrosis, although the limited availability of FibroScan data in our cohort precluded meaningful comparisons between these non-invasive markers.

The potential clinical role of liraglutide in CGL should be considered in relation to, but not as a substitute for, leptin replacement therapy. Metreleptin directly addresses the profound leptin deficiency that characterizes GL and therefore represents a disease-specific treatment. In contrast, liraglutide does not correct leptin deficiency but may provide complementary metabolic benefits through GLP-1 receptor activation, including improved glycemic control, appetite reduction, and potentially favorable effects on body weight and lipid metabolism. These effects may be particularly relevant in settings where disease-specific therapy is unavailable or access is limited. However, liraglutide also has important limitations, including the lack of disease-specific action, limited evidence supporting its use in CGL, and gastrointestinal adverse effects that may affect treatment tolerability. Importantly, because none of our participants had received leptin replacement and our study did not evaluate combined therapy, our findings cannot establish whether liraglutide offers additional benefit when used alongside disease-specific treatment or whether it can be considered an alternative to leptin replacement. Prospective studies are therefore needed to determine the optimal role of GLP-1RAs, including their potential use as adjunctive therapy to leptin replacement.

Beyond its potential metabolic effects, the accessibility and cost of liraglutide may also be relevant to its potential clinical use. Although oral and once-weekly injectable GLP-1RAs are increasingly available and have well-established benefits in the treatment of diabetes and obesity, their cost may limit access in some settings. Liraglutide, despite requiring daily administration, has demonstrated efficacy and safety across a range of metabolic conditions and may offer a potentially more accessible option than some newer GLP-1RAs in certain healthcare settings. Thus, where access or cost may limit the use of other GLP-1RAs, liraglutide may warrant consideration as a potential adjunctive metabolic therapy, rather than as a replacement for disease-specific leptin replacement.

This study has some limitations. Firstly, the very small sample size, inherent to the rarity of CGL, substantially limited the statistical power of the analyses and increased the risk of false positives and false negatives. Therefore, the reported p-values ​​should be interpreted with caution and should not be considered definitive evidence of treatment efficacy. Instead, the findings should be viewed as exploratory and hypothesis-generating, justifying confirmation in larger prospective studies.

Another important limitation is the absence of a concurrent control group, which is inherent to the case series design and precludes causal inference. Consequently, the observed changes in metabolic parameters cannot be attributed exclusively to liraglutide treatment, as they may also reflect regression to the mean, the natural variability of congenital generalized lipodystrophy, or unmeasured changes in lifestyle behaviors, including dietary intake, despite participants being instructed to maintain their usual diet throughout the study.

Moreover, the 12-week treatment period was relatively short to adequately evaluate changes in body composition, particularly skeletal muscle mass and the potential development or progression of sarcopenia. In addition, DEXA assessments could not be performed in all participants, further limiting the evaluation of changes in body composition. Likewise, the short follow-up precluded robust assessment of hepatic outcomes, especially changes in liver fibrosis, which typically require longer observation periods. The interpretation of hepatic outcomes was also limited by the fact that FibroScan examinations could not be performed in all participants, resulting in a limited number of measurements for this assessment. Similarly, satiety was assessed using a visual analogue scale (VAS), a subjective instrument that is susceptible to intraindividual variability and may not fully capture changes in appetite over time. Therefore, the effects of liraglutide on body composition, hepatic disease progression, and satiety should be interpreted with caution and confirmed in larger, long-term prospective studies.

An additional limitation is the genetic heterogeneity of the cases. Although most participants had CGL subtype 1 (CGL1) due to *AGPAT2* variants (7/8), one participant had CGL subtype 2 (CGL2) associated with a *BSCL2* variant. These genetic subtypes differ in disease pathophysiology, adipose tissue distribution, and metabolic severity, with CGL2 generally exhibiting a more severe phenotype (1). These differences may influence the response to GLP-1RAs. Given the very small sample size, genotype-specific analyses were not feasible, and our findings primarily reflect the response of individuals with CGL1. Future studies including larger cohorts are needed to determine whether therapeutic responses differ according to the underlying genetic subtype of CGL.

Furthermore, liraglutide was associated with adverse events consistent with its established safety profile. However, the relatively short follow-up period limits the assessment of its long-term safety and tolerability. Notably, treatment discontinuation due to gastrointestinal adverse events occurred in 37.5% of participants, a proportion that appears higher than that reported in liraglutide trials involving individuals with obesity or type 2 diabetes (26, 28). Although meaningful comparisons are limited by the small sample size, this observation raises the possibility that patients with CGL may be particularly susceptible to the gastrointestinal effects of GLP-1RAs. Profound leptin deficiency, a hallmark of CGL, results in severe hyperphagia and may alter neuroendocrine pathways involved in appetite regulation and gastrointestinal physiology, potentially influencing treatment tolerability. Our dose-escalation protocol followed the approved prescribing information, with weekly escalation to a target dose of 1.8 mg/day according to tolerability; however, all treatment discontinuations occurred during the titration phase, before the target dose was achieved. Although no definitive conclusions can be drawn, these findings suggest that a slower and more individualized titration strategy may improve tolerability and treatment adherence in this population. Whether the increased frequency of gastrointestinal adverse events reflects disease-specific mechanisms, the titration schedule, or both remains uncertain and should be investigated in future prospective studies.

Despite these limitations, our findings indicate that liraglutide may improve metabolic outcomes in patients with CGL, suggesting that GLP-1RAs could represent a therapeutic option in this population.

## Conclusion

This first study of liraglutide in CGL provides an exploratory signal of improved glycemic control and reduced insulin requirements in patients with this rare, heterogeneous, and clinically challenging condition. However, the substantial treatment discontinuation rate (3/8 participants; 37.5%) highlights an important limitation regarding treatment tolerability. Larger, controlled, and longer-term studies are needed to confirm the potential effects of liraglutide on glycemic control and insulin requirements, as well as to evaluate its effects on satiety, body composition, and liver disease. Future studies should also investigate whether liraglutide may provide additional metabolic benefits when used in combination with metreleptin.

## Data Availability

The original contributions presented in the study are included in the article, further inquiries can be directed to the corresponding author.

## References

[1] Akinci B Meral R Oral EA . Phenotypic and genetic characteristics of lipodystrophy: pathophysiology, metabolic abnormalities, and comorbidities. Curr Diabetes Rep. (2018) 18:143. doi: 10.1007/s11892-018-1099-9 30406415

[2] Rochford JJ . When adipose tissue lets you down: understanding the functions of genes disrupted in lipodystrophy. Diabetes. (2022) 71:589–98. doi: 10.2337/dbi21-0006 35316838

[3] Lima JG Nobrega LHC Lima NN Dos Santos MCF Silva PHD Baracho MFP . Causes of death in patients with Berardinelli-Seip congenital generalized lipodystrophy. PloS One. (2018) 13:e0199052. doi: 10.1371/journal.pone.0199052 29883474 PMC5993255

[4] Magre J Delepine M Khallouf E Gedde-Dahl T Jr Van Maldergem L Sobel E . Identification of the gene altered in Berardinelli-Seip congenital lipodystrophy on chromosome 11q13. Nat Genet. (2001) 28:365–70. doi: 10.1038/ng585 11479539

[5] Brown RJ Oral EA Cochran E Araujo-Vilar D Savage DB Long A . Long-term effectiveness and safety of metreleptin in patients with generalized lipodystrophy. Endocrine. (2018) 60:479–89. doi: 10.1007/s12020-018-1589-1 29644599 PMC5936645

[6] Sued Leão I Rodrigues Dantas J Galvão S Rodacki M Zajdenverg L . Long-term clinical experience with metreleptin in a Brazilian patient with congenital generalized lipodystrophy type 2. JCEM Case Rep. (2025) 3:luaf185. doi: 10.1210/jcemcr/luaf185 40860570 PMC12371232

[7] Muller TD Finan B Bloom SR D'Alessio D Drucker DJ Flatt PR . Glucagon-like peptide-1 (GLP-1). Mol Metab. (2019) 30:72–130. doi: 10.1016/j.molmet.2019.09.010 PMC6812410 31767182

[8] Akinci B Oral EA Neidert A Rus D Brown RJ Longo M . Comorbidities and survival in patients with lipodystrophy: an international chart review study. J Clin Endocrinol Metab. (2019) 104:5120–35. doi: 10.1210/jc.2018-02730 31314093 PMC6760298

[9] American Diabetes Association Professional Practice Committee . Cardiovascular disease and risk management: Standards of Care in Diabetes—2025. Diabetes Care. (2025) 48:S207–38. doi: 10.2337/dc25-S010 39651970 PMC11635050

[10] Hussain I Patni N Garg A . Lipodystrophies, dyslipidaemias and atherosclerotic cardiovascular disease. Pathology. (2019) 51:202–12. doi: 10.1016/j.pathol.2018.11.004 30595509 PMC6402807

[11] Oliveira J Lau E Carvalho D Freitas P . Glucagon-like peptide-1 analogues as a therapeutic option for severe insulin resistance in lipodystrophic syndromes: two case reports. J Med Case Rep. (2017) 11:12. doi: 10.1186/s13256-016-1175-1 28086952 PMC5237351

[12] Banning F Rottenkolber M Freibothe I Seissler J Lechner A . Insulin secretory defect in familial partial lipodystrophy type 2 and long-term treatment with a glucagon-like peptide-1 receptor agonist. Diabetes Med. (2017) 34:1792–4. doi: 10.1111/dme.13527 29044799

[13] Foss-Freitas MC Imam S Neidert A Dill Gomes A Broome DT Oral EA . Efficacy and safety of glucagon-like peptide-1 receptor agonists in patients with familial partial lipodystrophy. Diabetes Care. (2024) 47:653–9. doi: 10.2337/dc23-1614 PMC10973902 38300898

[14] Lebenthal Y Halperin S Interator H Brener A Brown RJ . Oral glucagon-like peptide 1 analog as an adjuvant therapy in generalized lipodystrophy. Diabetes Care. (2025) 48:e71–3. doi: 10.2337/dc25-0272 40105772 PMC12034891

[15] Roumane A McIlroy GD Sommer N Han W Heisler LK Rochford JJ . GLP-1 receptor agonist improves metabolic disease in a preclinical model of lipodystrophy. Front Endocrinol. (2024) 15:1379228. doi: 10.1101/2023.09.01.555852 38745956 PMC11091257

[16] Meral R Celik Guler M Kaba D Prativadi J Frontera ED Foss-Freitas MC . Metabolic improvements with tirzepatide in lipodystrophy. Diabetes Care. (2025) 48:756–62. doi: 10.2337/dc24-2408 40063619 PMC12034899

[17] Flint A Raben A Blundell JE Astrup A . Reproducibility, power and validity of visual analogue scales in assessment of appetite sensations in single test meal studies. Int J Obes Related Metab Disord. (2000) 24:38–48. doi: 10.1038/sj.ijo.0801083 10702749

[18] Kristensen SL Rørth R Jhund PS Docherty KF Sattar N Preiss D . Cardiovascular, mortality, and kidney outcomes with glucagon-like peptide-1 receptor agonists in patients with type 2 diabetes: a systematic review and meta-analysis. Lancet Diabetes Endocrinol. (2019) 7:776–85. doi: 10.1016/s2213-8587(19)30249-9 31422062

[19] Sattar N Lee MMY Kristensen SL Branch KRH Del Prato S Khurmi NS . Cardiovascular, mortality, and kidney outcomes with glucagon-like peptide-1 receptor agonists in randomized trials. Lancet Diabetes Endocrinol. (2021) 9:653–62. doi: 10.1016/S2213-8587(21)00203-5 34425083

[20] McDuffie JR Riggs PA Calis KA Freedman RJ Oral EA DePaoli AM . Effects of exogenous leptin on satiety and satiation in patients with lipodystrophy. J Clin Endocrinol Metab. (2004) 89:4258–63. doi: 10.1210/jc.2003-031868 15356018 PMC2266890

[21] Oral EA Simha V Ruiz E Andewelt A Premkumar A Snell P . Leptin replacement therapy for lipodystrophy. N Engl J Med. (2002) 346:570–8. doi: 10.1056/nejmoa012437 11856796

[22] Brown RJ Valencia A Startzell M Cochran E Walter PJ Garraffo HM . Metreleptin-mediated improvements in insulin sensitivity are independent of food intake in humans with lipodystrophy. J Clin Invest. (2018) 128:3504–16. doi: 10.1172/jci95476 29723161 PMC6063484

[23] Rupp AC Tomlinson AJ Affinati AH Yacawych WT Duensing AM True C . Suppression of food intake by Glp1r/Lepr coexpressing neurons prevents obesity in mouse models. J Clin Invest. (2023) 133:e157515. doi: 10.1172/JCI157515 PMC10541203 37581939

[24] Polex-Wolf J Deibler K Hogendorf WFJ Bau S Glendorf T Stidsen CE . Glp1r-Lepr coexpressing neurons modulate suppression of food intake and body weight by a GLP-1/leptin dual agonist. Sci Transl Med. (2024) 16:eadk4908. doi: 10.1126/scitranslmed.adk4908 39630884 PMC12967300

[25] Brown RJ Araujo-Vilar D Cheung PT Dunger D Garg A Jack M . The diagnosis and management of lipodystrophy syndromes: a multisociety practice guideline. J Clin Endocrinol Metab. (2016) 101:4500–11. doi: 10.1210/jc.2016-2466 27710244 PMC5155679

[26] Ten S Bhangoo A Buettner C . Tirzepatide for congenital generalized lipodystrophy. N Engl J Med. (2025) 392:717–9. doi: 10.1056/nejmc2413871 39886995

[27] le Roux CW Astrup A Fujioka K Greenway F Halpern A Krempf M . Three years of liraglutide versus placebo for type 2 diabetes risk reduction and weight management in individuals with prediabetes. Lancet. (2017) 389:1399–409. doi: 10.1016/s0140-6736(17)30069-7 28237263

[28] Rubino D Abrahamsson N Davies M Hesse D Greenway FL Jensen C . Effect of continued weekly subcutaneous semaglutide vs placebo on weight loss maintenance in adults with overweight or obesity: the STEP 4 randomized clinical trial. JAMA. (2021) 325:1414–25. doi: 10.1001/jama.2021.3224 33755728 PMC7988425

[29] Pi-Sunyer X Astrup A Fujioka K Greenway F Halpern A Krempf M . A randomized, controlled trial of liraglutide 3.0 mg for weight management. N Engl J Med. (2015) 373:11–22. doi: 10.1056/nejmoa1411892 26132939

[30] Wilding JPH Batterham RL Davies M Van Gaal LF Kandler K Konakli K . Weight regain and cardiometabolic effects after withdrawal of semaglutide: the STEP 1 trial extension. Diabetes Obes Metab. (2022) 24:1553–64. doi: 10.1111/dom.14725 35441470 PMC9542252

[31] Aronne LJ Sattar N Horn DB Bays HE Wharton S Lin WY . Continued treatment with tirzepatide for maintenance of weight reduction in adults with obesity: the SURMOUNT-4 randomized clinical trial. JAMA. (2024) 331:38–48. doi: 10.1001/jama.2023.24945 38078870 PMC10714284

[32] Gilden AH Catenacci VA Taormina JM . Obesity. Ann Intern Med. (2024) 177:ITC65–80. doi: 10.7326/AITC202405210 38739920

[33] Sumithran P Prendergast LA Delbridge E Purcell K Shulkes A Kriketos A . Long-term persistence of hormonal adaptations to weight loss. N Engl J Med. (2011) 365:1597–604. doi: 10.1056/NEJMoa1105816 22029981

[34] Quarenghi M Capelli S Galligani G Giana A Preatoni G Turri Quarenghi R . Weight regain after liraglutide, semaglutide or tirzepatide interruption: a narrative review of randomized studies. J Clin Med. (2025) 14:3791. doi: 10.3390/jcm14113791 40507553 PMC12155999

[35] Tan Y Zhen Q Ding X Shen T Liu F Wang Y . Association between use of liraglutide and liver fibrosis in patients with type 2 diabetes. Front Endocrinol (Lausanne). (2022) 13:935180. doi: 10.3389/fendo.2022.935180 36034438 PMC9399467

[36] Bołdys A Borówka M Bułdak Ł Okopień B . Impact of short-term liraglutide therapy on non-invasive markers of liver fibrosis in patients with MASLD. Metabolites. (2025) 15:510. doi: 10.3390/metabo15080510 40863129. Published 2025 Jul 31. PMC12388381

[37] Wood M Kennedy AG Khan S Hitt JR Davis K Reddy SS . Impact of GLP-1 receptor agonist use in patients with steatotic liver disease and type 2 diabetes: A retrospective cohort study. J Pharm Pract. (2024) 37:1297–302. doi: 10.1177/08971900241253661 38720191

[38] Kumar S Lahooti A Hajifathalian K Criteli B Hassan A Johnson KE . Prospective, matched case–control study of endoscopic sleeve gastroplasty and semaglutide in patients with metabolic dysfunction-associated steatotic liver disease at one year. Dig Dis Sci. (2025) 70:3922–8. doi: 10.1007/s10620-025-09218-1 40629230

[39] Dubin RL Heymsfield SB Ravussin E Greenway FL . Glucagon-like peptide-1 receptor agonist-based agents and weight loss composition: Filling the gaps. Diabetes Obes Metab. (2024) 26:5503–18. doi: 10.1111/dom.15913 39344838

